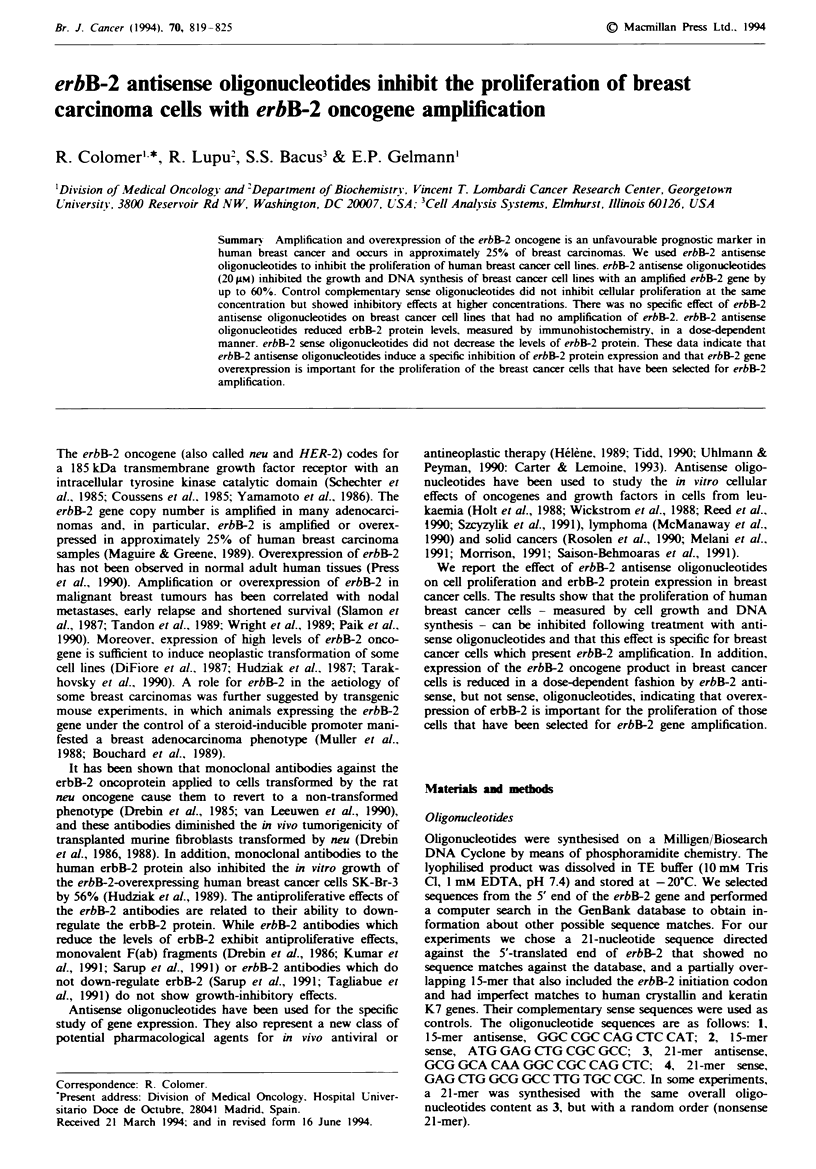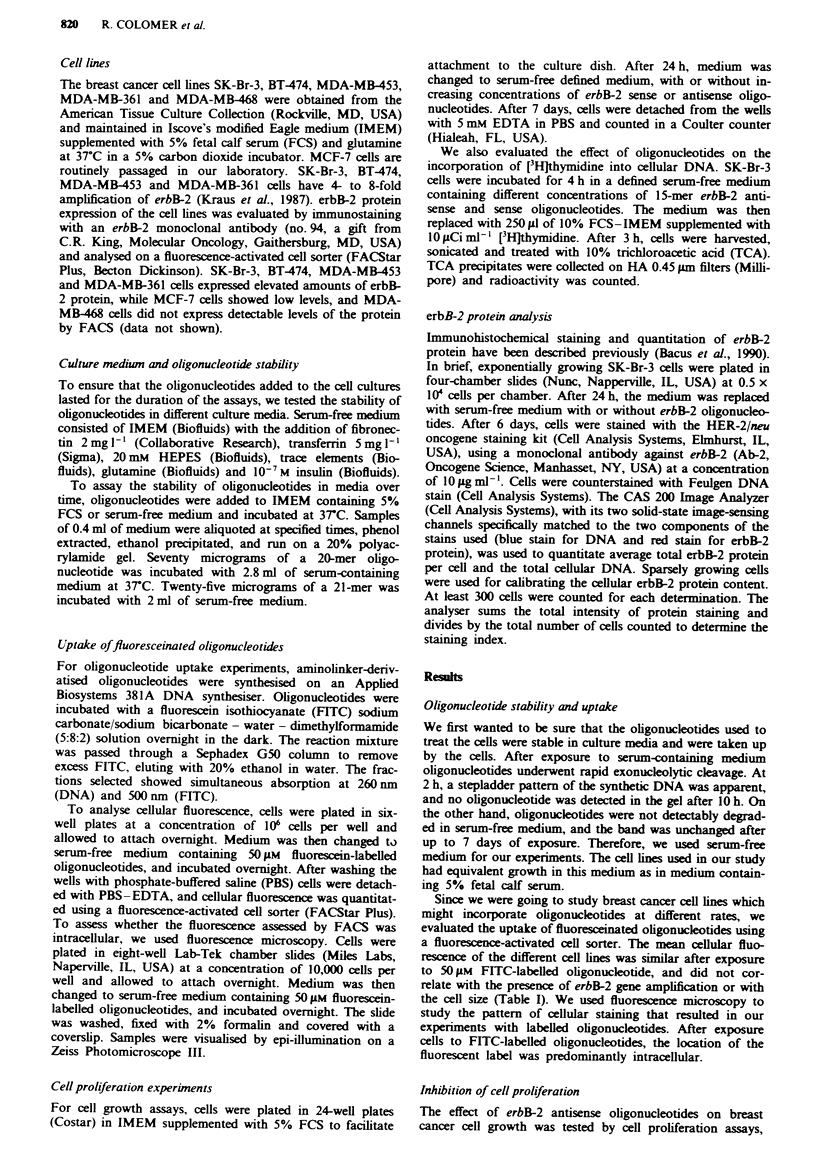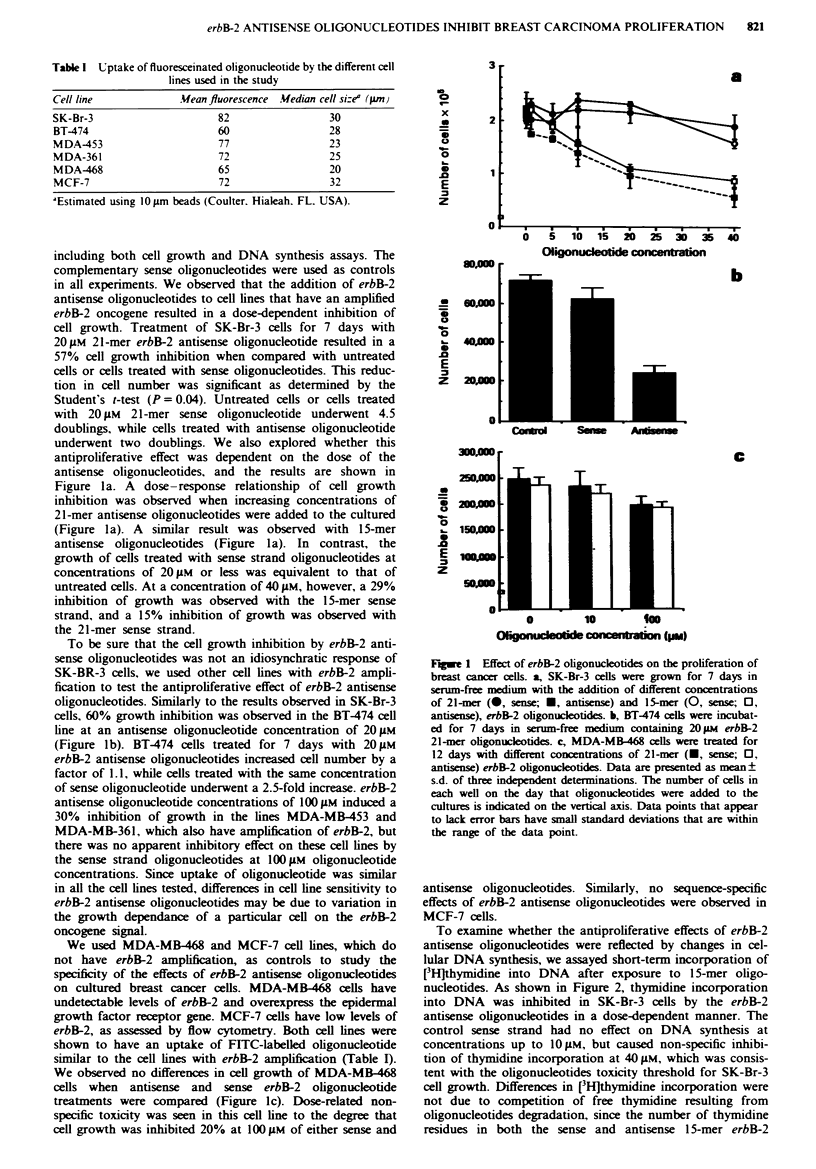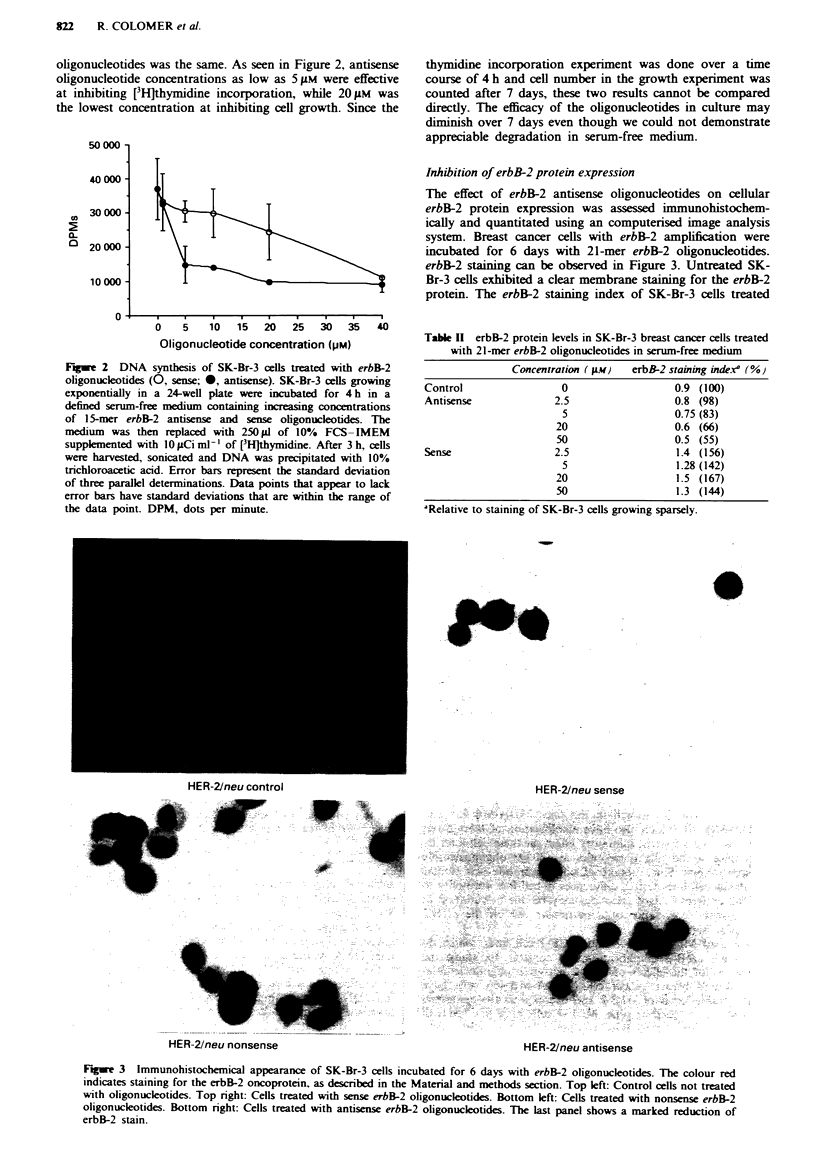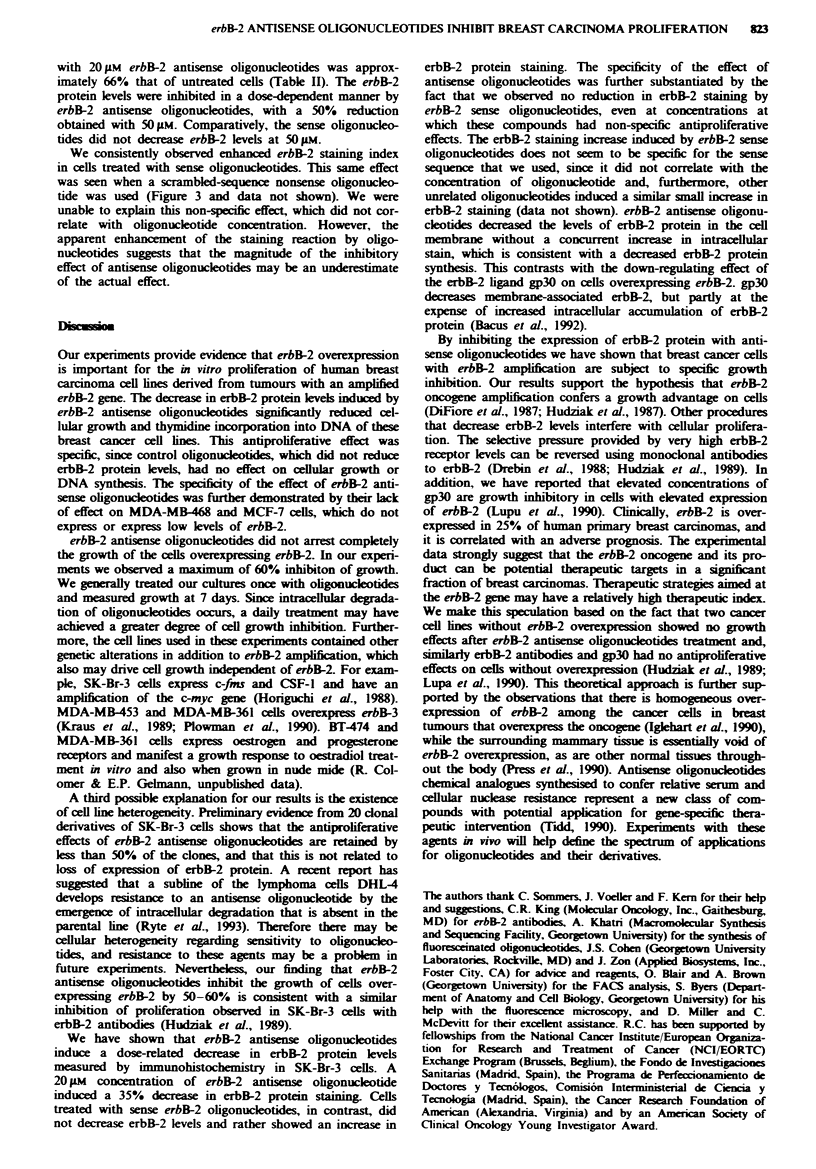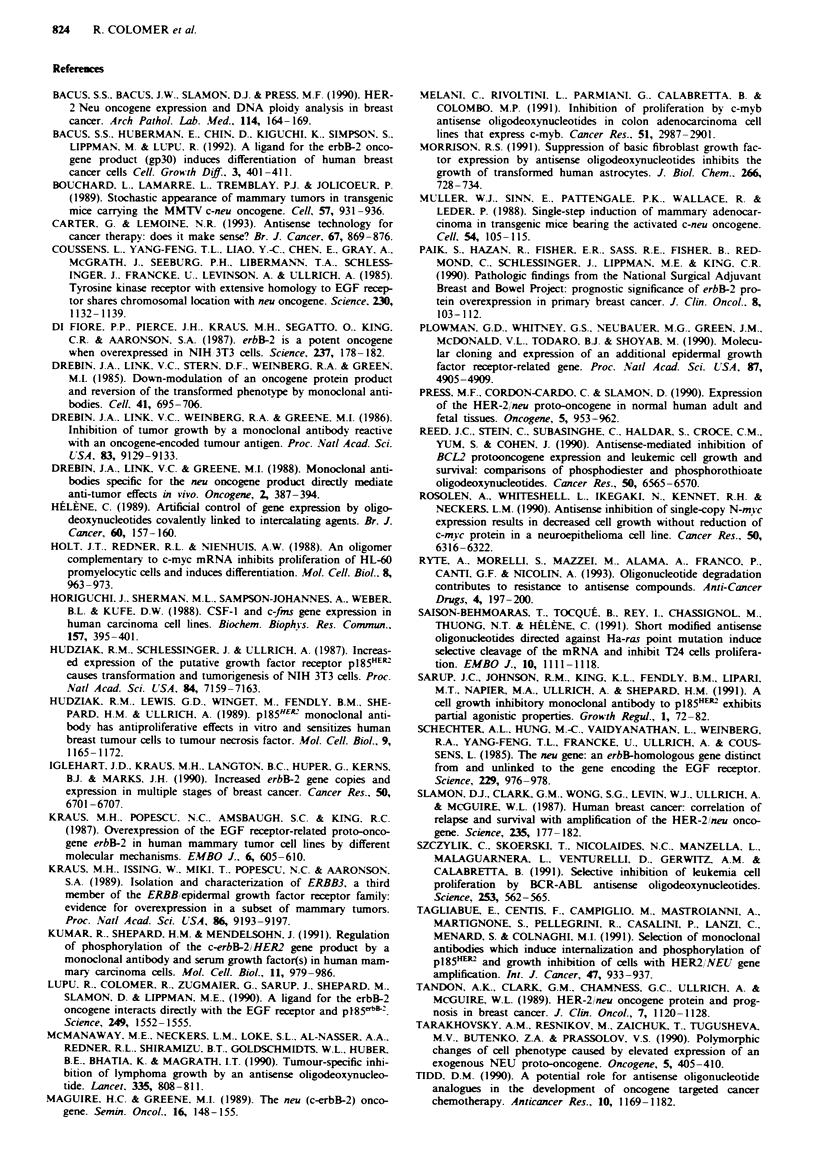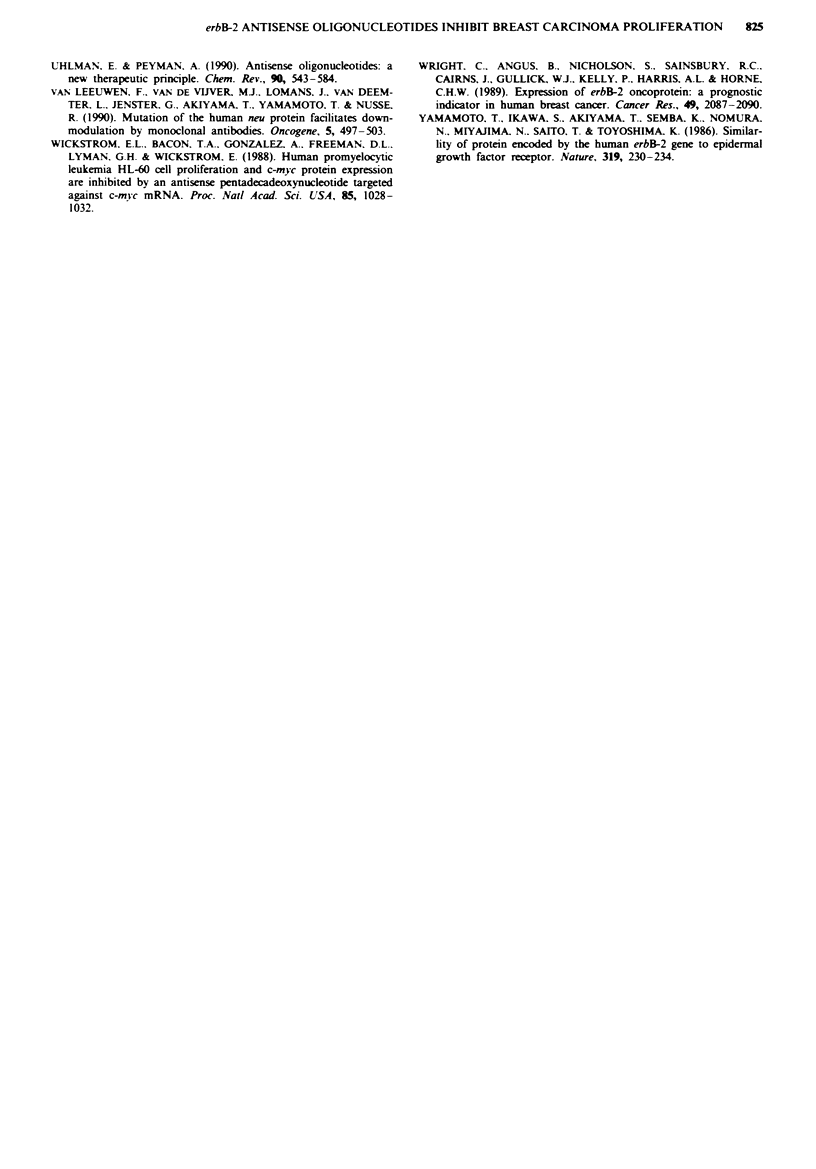# erbB-2 antisense oligonucleotides inhibit the proliferation of breast carcinoma cells with erbB-2 oncogene amplification.

**DOI:** 10.1038/bjc.1994.405

**Published:** 1994-11

**Authors:** R. Colomer, R. Lupu, S. S. Bacus, E. P. Gelmann

**Affiliations:** Division of Medical Oncology, Vincent T. Lombardi Cancer Research Center, Georgetown University, Washington, DC 20007.

## Abstract

**Images:**


### erbB-2 antisense oligonucleotides inhibit the proliferation of breast carcinoma cells with erbB-2 oncogene amplification

R. Colomer[1,*], R. Lupu[2], S.S. Bacus[3] & E.P. Gelmann[1]

[1]Division of Medical Oncology and [2]Department of Biochemistry, Vincent T. Lombardi Cancer Research Center, Georgetown University, 3800 Reservoir Rd NW, Washington, DC 20007, USA; [3]Cell Analysis Systems, Elmhurst, Illinois 60126, USA

**Summary** Amplification and overexpression of the *erb*B-2 oncogene is an unfavourable prognostic marker in human breast cancer and occurs in approximately 25% of breast carcinomas. We used *erb*B-2 antisense oligonucleotides to inhibit the proliferation of human breast cancer cell lines. *erb*B-2 antisense oligonucleotides (20 μM) inhibited the growth and DNA synthesis of breast cancer cell lines with an amplified *erb*B-2 gene by up to 60%. Control complementary sense oligonucleotides did not inhibit cellular proliferation at the same concentration but showed inhibitory effects at higher concentrations. There was no specific effect of *erb*B-2 antisense oligonucleotides on breast cancer cell lines that had no amplification of *erb*B-2. *erb*B-2 antisense oligonucleotides reduced erbB-2 protein levels, measured by immunohistochemistry, in a dose-dependent manner. *erb*B-2 sense oligonucleotides did not decrease the levels of *erb*B-2 protein. These data indicate that *erb*B-2 antisense oligonucleotides induce a specific inhibition of *erb*B-2 protein expression and that *erb*B-2 gene overexpression is important for the proliferation of the breast cancer cells that have been selected for *erb*B-2 amplification.

The *erb*B-2 oncogene (also called *neu* and *HER-2*) codes for a 185 kDa transmembrane growth factor receptor with an intracellular tyrosine kinase catalytic domain (Schechter *et al.*, 1985; Coussens *et al.*, 1985; Yamamoto *et al.*, 1986). The *erb*B-2 gene copy number is amplified in many adenocarcinomas and, in particular, *erb*B-2 is amplified or overexpressed in approximately 25% of human breast carcinoma samples (Maguire & Greene, 1989). Overexpression of *erb*B-2 has not been observed in normal adult human tissues (Press *et al.*, 1990). Amplification or overexpression of *erb*B-2 in malignant breast tumours has been correlated with nodal metastases, early relapse and shortened survival (Slamon *et al.*, 1987; Tandon *et al.*, 1989; Wright *et al.*, 1989; Paik *et al.*, 1990). Moreover, expression of high levels of *erb*B-2 oncogene is sufficient to induce neoplastic transformation of some cell lines (DiFiore *et al.*, 1987; Hudziak *et al.*, 1987; Tarakhovsky *et al.*, 1990). A role for *erb*B-2 in the aetiology of some breast carcinomas was further suggested by transgenic mouse experiments, in which animals expressing the *erb*B-2 gene under the control of a steroid-inducible promoter manifested a breast adenocarcinoma phenotype (Muller *et al.*, 1988; Bouchard *et al.*, 1989).

It has been shown that monoclonal antibodies against the erbB-2 oncoprotein applied to cells transformed by the rat *neu* oncogene cause them to revert to a non-transformed phenotype (Drebin *et al.*, 1985; van Leeuwen *et al.*, 1990), and these antibodies diminished the *in vivo* tumorigenicity of transplanted murine fibroblasts transformed by *neu* (Drebin *et al.*, 1986, 1988). In addition, monoclonal antibodies to the human erbB-2 protein also inhibited the *in vitro* growth of the *erb*B-2-overexpressing human breast cancer cells SK-Br-3 by 56% (Hudziak *et al.*, 1989). The antiproliferative effects of the *erb*B-2 antibodies are related to their ability to down-regulate the erbB-2 protein. While *erb*B-2 antibodies which reduce the levels of erbB-2 exhibit antiproliferative effects, monovalent F(ab) fragments (Drebin *et al.*, 1986; Kumar *et al.*, 1991; Sarup *et al.*, 1991) or *erb*B-2 antibodies which do not down-regulate erbB-2 (Sarup *et al.*, 1991; Tagliabue *et al.*, 1991) do not show growth-inhibitory effects.

Antisense oligonucleotides have been used for the specific study of gene expression. They also represent a new class of potential pharmacological agents for *in vivo* antiviral or antineoplastic therapy (Hélène, 1989; Tidd, 1990; Uhlmann & Peyman, 1990: Carter & Lemoine, 1993). Antisense oligonucleotides have been used to study the *in vitro* cellular effects of oncogenes and growth factors in cells from leukaemia (Holt *et al.*, 1988; Wickstrom *et al.*, 1988; Reed *et al.*, 1990; Szczyzylik *et al.*, 1991), lymphoma (McManaway *et al.*, 1990) and solid cancers (Rosolen *et al.*, 1990; Melani *et al.*, 1991; Morrison, 1991; Saison-Behmoaras *et al.*, 1991).

We report the effect of *erb*B-2 antisense oligonucleotides on cell proliferation and erbB-2 protein expression in breast cancer cells. The results show that the proliferation of human breast cancer cells – measured by cell growth and DNA synthesis – can be inhibited following treatment with antisense oligonucleotides and that this effect is specific for breast cancer cells which present *erb*B-2 amplification. In addition, expression of the *erb*B-2 oncogene product in breast cancer cells is reduced in a dose-dependent fashion by *erb*B-2 antisense, but not sense, oligonucleotides, indicating that overexpression of erbB-2 is important for the proliferation of those cells that have been selected for *erb*B-2 gene amplification.

### Materials and methods

### Oligonucleotides

Oligonucleotides were synthesised on a Milligen/Biosearch DNA Cyclone by means of phosphoramidite chemistry. The lyophilised product was dissolved in TE buffer (10 mM Tris Cl, 1 mM EDTA, pH 7.4) and stored at −20°C. We selected sequences from the 5′ end of the *erb*B-2 gene and performed a computer search in the GenBank database to obtain information about other possible sequence matches. For our experiments we chose a 21-nucleotide sequence directed against the 5′-translated end of *erb*B-2 that showed no sequence matches against the database, and a partially overlapping 15-mer that also included the *erb*B-2 initiation codon and had imperfect matches to human crystallin and keratin K7 genes. Their complementary sense sequences were used as controls. The oligonucleotide sequences are as follows: **1**, 15-mer antisense, GGC CGC CAG CTC CAT; **2**, 15-mer sense, ATG GAG CTG CGC GCC; **3**, 21-mer antisense, GCG GCA CAA GGC CGC CAG CTC; **4**, 21-mer sense, GAG CTG GCG GCC TTG TGC CGC. In some experiments, a 21-mer was synthesised with the same overall oligonucleotides content as **3**, but with a random order (nonsense 21-mer).

Correspondence: R. Colomer.
*Present address: Division of Medical Oncology, Hospital Universitario Doce de Octubre, 28041 Madrid, Spain.
Received 21 March 1994; and in revised form 16 June 1994.

*Cell lines*

The breast cancer cell lines SK-Br-3, BT-474, MDA-MB-453, MDA-MB-361 and MDA-MB-468 were obtained from the American Tissue Culture Collection (Rockville, MD, USA) and maintained in Iscove's modified Eagle medium (IMEM) supplemented with 5% fetal calf serum (FCS) and glutamine at 37°C in a 5% carbon dioxide incubator. MCF-7 cells are routinely passaged in our laboratory. SK-Br-3, BT-474, MDA-MB-453 and MDA-MB-361 cells have 4- to 8-fold amplification of *erb*B-2 (Kraus *et al.*, 1987). erbB-2 protein expression of the cell lines was evaluated by immunostaining with an *erb*B-2 monoclonal antibody (no. 94, a gift from C.R. King, Molecular Oncology, Gaithersburg, MD, USA) and analysed on a fluorescence-activated cell sorter (FACStar Plus, Becton Dickinson). SK-Br-3, BT-474, MDA-MB-453 and MDA-MB-361 cells expressed elevated amounts of erbB-2 protein, while MCF-7 cells showed low levels, and MDA-MB-468 cells did not express detectable levels of the protein by FACS (data not shown).

*Culture medium and oligonucleotide stability*

To ensure that the oligonucleotides added to the cell cultures lasted for the duration of the assays, we tested the stability of oligonucleotides in different culture media. Serum-free medium consisted of IMEM (Biofluids) with the addition of fibronectin 2 mg $l^{-1}$ (Collaborative Research), transferrin 5 mg $l^{-1}$ (Sigma), 20 mM HEPES (Biofluids), trace elements (Biofluids), glutamine (Biofluids) and $10^{-7}$ M insulin (Biofluids).

To assay the stability of oligonucleotides in media over time, oligonucleotides were added to IMEM containing 5% FCS or serum-free medium and incubated at 37°C. Samples of 0.4 ml of medium were aliquoted at specified times, phenol extracted, ethanol precipitated, and run on a 20% polyacrylamide gel. Seventy micrograms of a 20-mer oligonucleotide was incubated with 2.8 ml of serum-containing medium at 37°C. Twenty-five micrograms of a 21-mer was incubated with 2 ml of serum-free medium.

*Uptake of fluoresceinated oligonucleotides*

For oligonucleotide uptake experiments, aminolinker-derivatised oligonucleotides were synthesised on an Applied Biosystems 381A DNA synthesiser. Oligonucleotides were incubated with a fluorescein isothiocyanate (FITC) sodium carbonate/sodium bicarbonate – water – dimethylformamide (5:8:2) solution overnight in the dark. The reaction mixture was passed through a Sephadex G50 column to remove excess FITC, eluting with 20% ethanol in water. The fractions selected showed simultaneous absorption at 260 nm (DNA) and 500 nm (FITC).

To analyse cellular fluorescence, cells were plated in six-well plates at a concentration of $10^6$ cells per well and allowed to attach overnight. Medium was then changed to serum-free medium containing 50 μM fluorescein-labelled oligonucleotides, and incubated overnight. After washing the wells with phosphate-buffered saline (PBS) cells were detached with PBS–EDTA, and cellular fluorescence was quantitated using a fluorescence-activated cell sorter (FACStar Plus). To assess whether the fluorescence assessed by FACS was intracellular, we used fluorescence microscopy. Cells were plated in eight-well Lab-Tek chamber slides (Miles Labs, Naperville, IL, USA) at a concentration of 10,000 cells per well and allowed to attach overnight. Medium was then changed to serum-free medium containing 50 μM fluorescein-labelled oligonucleotides, and incubated overnight. The slide was washed, fixed with 2% formalin and covered with a coverslip. Samples were visualised by epi-illumination on a Zeiss Photomicroscope III.

*Cell proliferation experiments*

For cell growth assays, cells were plated in 24-well plates (Costar) in IMEM supplemented with 5% FCS to facilitate attachment to the culture dish. After 24 h, medium was changed to serum-free defined medium, with or without increasing concentrations of *erb*B-2 sense or antisense oligonucleotides. After 7 days, cells were detached from the wells with 5 mM EDTA in PBS and counted in a Coulter counter (Hialeah, FL, USA).

We also evaluated the effect of oligonucleotides on the incorporation of [$^3$H]thymidine into cellular DNA. SK-Br-3 cells were incubated for 4 h in a defined serum-free medium containing different concentrations of 15-mer *erb*B-2 antisense and sense oligonucleotides. The medium was then replaced with 250 μl of 10% FCS–IMEM supplemented with 10 μCi $ml^{-1}$ [$^3$H]thymidine. After 3 h, cells were harvested, sonicated and treated with 10% trichloroacetic acid (TCA). TCA precipitates were collected on HA 0.45 μm filters (Millipore) and radioactivity was counted.

*erbB-2 protein analysis*

Immunohistochemical staining and quantitation of *erb*B-2 protein have been described previously (Bacus *et al.*, 1990). In brief, exponentially growing SK-Br-3 cells were plated in four-chamber slides (Nunc, Nappperville, IL, USA) at 0.5 × $10^4$ cells per chamber. After 24 h, the medium was replaced with serum-free medium with or without *erb*B-2 oligonucleotides. After 6 days, cells were stained with the HER-2/*neu* oncogene staining kit (Cell Analysis Systems, Elmhurst, IL, USA), using a monoclonal antibody against *erb*B-2 (Ab-2, Oncogene Science, Manhasset, NY, USA) at a concentration of 10 μg $ml^{-1}$. Cells were counterstained with Feulgen DNA stain (Cell Analysis Systems). The CAS 200 Image Analyzer (Cell Analysis Systems), with its two solid-state image-sensing channels specifically matched to the two components of the stains used (blue stain for DNA and red stain for erbB-2 protein), was used to quantitate average total erbB-2 protein per cell and the total cellular DNA. Sparsely growing cells were used for calibrating the cellular erbB-2 protein content. At least 300 cells were counted for each determination. The analyser sums the total intensity of protein staining and divides by the total number of cells counted to determine the staining index.

### Results

*Oligonucleotide stability and uptake*

We first wanted to be sure that the oligonucleotides used to treat the cells were stable in culture media and were taken up by the cells. After exposure to serum-containing medium oligonucleotides underwent rapid exonucleolytic cleavage. At 2 h, a stepladder pattern of the synthetic DNA was apparent, and no oligonucleotide was detected in the gel after 10 h. On the other hand, oligonucleotides were not detectably degraded in serum-free medium, and the band was unchanged after up to 7 days of exposure. Therefore, we used serum-free medium for our experiments. The cell lines used in our study had equivalent growth in this medium as in medium containing 5% fetal calf serum.

Since we were going to study breast cancer cell lines which might incorporate oligonucleotides at different rates, we evaluated the uptake of fluoresceinated oligonucleotides using a fluorescence-activated cell sorter. The mean cellular fluorescence of the different cell lines was similar after exposure to 50 μM FITC-labelled oligonucleotide, and did not correlate with the presence of *erb*B-2 gene amplification or with the cell size (Table I). We used fluorescence microscopy to study the pattern of cellular staining that resulted in our experiments with labelled oligonucleotides. After exposure cells to FITC-labelled oligonucleotides, the location of the fluorescent label was predominantly intracellular.

*Inhibition of cell proliferation*

The effect of *erb*B-2 antisense oligonucleotides on breast cancer cell growth was tested by cell proliferation assays,

**Table I** Uptake of fluoresceinated oligonucleotide by the different cell lines used in the study

| *Cell line* | *Mean fluorescence* | *Median cell size*[a] *(μm)* |
|---|---|---|
| SK-Br-3 | 82 | 30 |
| BT-474 | 60 | 28 |
| MDA-453 | 77 | 23 |
| MDA-361 | 72 | 25 |
| MDA-468 | 65 | 20 |
| MCF-7 | 72 | 32 |

[a]Estimated using 10 μm beads (Coulter, Hialeah, FL, USA).

including both cell growth and DNA synthesis assays. The complementary sense oligonucleotides were used as controls in all experiments. We observed that the addition of *erb*B-2 antisense oligonucleotides to cell lines that have an amplified *erb*B-2 oncogene resulted in a dose-dependent inhibition of cell growth. Treatment of SK-Br-3 cells for 7 days with 20 μM 21-mer *erb*B-2 antisense oligonucleotide resulted in a 57% cell growth inhibition when compared with untreated cells or cells treated with sense oligonucleotides. This reduction in cell number was significant as determined by the Student's *t*-test ($P = 0.04$). Untreated cells or cells treated with 20 μM 21-mer sense oligonucleotide underwent 4.5 doublings, while cells treated with antisense oligonucleotide underwent two doublings. We also explored whether this antiproliferative effect was dependent on the dose of the antisense oligonucleotides, and the results are shown in Figure 1a. A dose-response relationship of cell growth inhibition was observed when increasing concentrations of 21-mer antisense oligonucleotides were added to the cultured (Figure 1a). A similar result was observed with 15-mer antisense oligonucleotides (Figure 1a). In contrast, the growth of cells treated with sense strand oligonucleotides at concentrations of 20 μM or less was equivalent to that of untreated cells. At a concentration of 40 μM, however, a 29% inhibition of growth was observed with the 15-mer sense strand, and a 15% inhibition of growth was observed with the 21-mer sense strand.

To be sure that the cell growth inhibition by *erb*B-2 antisense oligonucleotides was not an idiosynchratic response of SK-BR-3 cells, we used other cell lines with *erb*B-2 amplification to test the antiproliferative effect of *erb*B-2 antisense oligonucleotides. Similarly to the results observed in SK-Br-3 cells, 60% growth inhibition was observed in the BT-474 cell line at an antisense oligonucleotide concentration of 20 μM (Figure 1b). BT-474 cells treated for 7 days with 20 μM *erb*B-2 antisense oligonucleotides increased cell number by a factor of 1.1, while cells treated with the same concentration of sense oligonucleotide underwent a 2.5-fold increase. *erb*B-2 antisense oligonucleotide concentrations of 100 μM induced a 30% inhibition of growth in the lines MDA-MB-453 and MDA-MB-361, which also have amplification of *erb*B-2, but there was no apparent inhibitory effect on these cell lines by the sense strand oligonucleotides at 100 μM oligonucleotide concentrations. Since uptake of oligonucleotide was similar in all the cell lines tested, differences in cell line sensitivity to *erb*B-2 antisense oligonucleotides may be due to variation in the growth dependance of a particular cell on the *erb*B-2 oncogene signal.

We used MDA-MB-468 and MCF-7 cell lines, which do not have *erb*B-2 amplification, as controls to study the specificity of the effects of *erb*B-2 antisense oligonucleotides on cultured breast cancer cells. MDA-MB-468 cells have undetectable levels of *erb*B-2 and overexpress the epidermal growth factor receptor gene. MCF-7 cells have low levels of *erb*B-2, as assessed by flow cytometry. Both cell lines were shown to have an uptake of FITC-labelled oligonucleotide similar to the cell lines with *erb*B-2 amplification (Table I). We observed no differences in cell growth of MDA-MB-468 cells when antisense and sense *erb*B-2 oligonucleotide treatments were compared (Figure 1c). Dose-related non-specific toxicity was seen in this cell line to the degree that cell growth was inhibited 20% at 100 μM of either sense and

**Figure 1** Effect of *erb*B-2 oligonucleotides on the proliferation of breast cancer cells. **a**, SK-Br-3 cells were grown for 7 days in serum-free medium with the addition of different concentrations of 21-mer (●, sense; ■, antisense) and 15-mer (O, sense; □, antisense), *erb*B-2 oligonucleotides. **b**, BT-474 cells were incubated for 7 days in serum-free medium containing 20 μM *erb*B-2 21-mer oligonucleotides. **c**, MDA-MB-468 cells were treated for 12 days with different concentrations of 21-mer (■, sense; □, antisense) *erb*B-2 oligonucleotides. Data are presented as mean ± s.d. of three independent determinations. The number of cells in each well on the day that oligonucleotides were added to the cultures is indicated on the vertical axis. Data points that appear to lack error bars have small standard deviations that are within the range of the data point.

antisense oligonucleotides. Similarly, no sequence-specific effects of *erb*B-2 antisense oligonucleotides were observed in MCF-7 cells.

To examine whether the antiproliferative effects of *erb*B-2 antisense oligonucleotides were reflected by changes in cellular DNA synthesis, we assayed short-term incorporation of [$^3$H]thymidine into DNA after exposure to 15-mer oligonucleotides. As shown in Figure 2, thymidine incorporation into DNA was inhibited in SK-Br-3 cells by the *erb*B-2 antisense oligonucleotides in a dose-dependent manner. The control sense strand had no effect on DNA synthesis at concentrations up to 10 μM, but caused non-specific inhibition of thymidine incorporation at 40 μM, which was consistent with the oligonucleotides toxicity threshold for SK-Br-3 cell growth. Differences in [$^3$H]thymidine incorporation were not due to competition of free thymidine resulting from oligonucleotides degradation, since the number of thymidine residues in both the sense and antisense 15-mer *erb*B-2

oligonucleotides was the same. As seen in Figure 2, antisense oligonucleotide concentrations as low as 5 μM were effective at inhibiting [$^{3}$H]thymidine incorporation, while 20 μM was the lowest concentration at inhibiting cell growth. Since the thymidine incorporation experiment was done over a time course of 4 h and cell number in the growth experiment was counted after 7 days, these two results cannot be compared directly. The efficacy of the oligonucleotides in culture may diminish over 7 days even though we could not demonstrate appreciable degradation in serum-free medium.

**Figure 2** DNA synthesis of SK-Br-3 cells treated with *erb*B-2 oligonucleotides (O, sense; ●, antisense). SK-Br-3 cells growing exponentially in a 24-well plate were incubated for 4 h in a defined serum-free medium containing increasing concentrations of 15-mer *erb*B-2 antisense and sense oligonucleotides. The medium was then replaced with 250 μl of 10% FCS-IMEM supplemented with 10 μCi ml$^{-1}$ of [$^{3}$H]thymidine. After 3 h, cells were harvested, sonicated and DNA was precipitated with 10% trichloroacetic acid. Error bars represent the standard deviation of three parallel determinations. Data points that appear to lack error bars have standard deviations that are within the range of the data point. DPM, dots per minute.

### *Inhibition of erbB-2 protein expression*

The effect of *erb*B-2 antisense oligonucleotides on cellular *erb*B-2 protein expression was assessed immunohistochemically and quantitated using an computerised image analysis system. Breast cancer cells with *erb*B-2 amplification were incubated for 6 days with 21-mer *erb*B-2 oligonucleotides. *erb*B-2 staining can be observed in Figure 3. Untreated SK-Br-3 cells exhibited a clear membrane staining for the *erb*B-2 protein. The *erb*B-2 staining index of SK-Br-3 cells treated

**Table II** erbB-2 protein levels in SK-Br-3 breast cancer cells treated with 21-mer *erb*B-2 oligonucleotides in serum-free medium

| | *Concentration ( μM )* | erb*B-2 staining index*[a] *( % )* |
|---|---|---|
| Control | 0 | 0.9 (100) |
| Antisense | 2.5 | 0.8 (98) |
| | 5 | 0.75 (83) |
| | 20 | 0.6 (66) |
| | 50 | 0.5 (55) |
| Sense | 2.5 | 1.4 (156) |
| | 5 | 1.28 (142) |
| | 20 | 1.5 (167) |
| | 50 | 1.3 (144) |

[a]Relative to staining of SK-Br-3 cells growing sparsely.

**Figure 3** Immunohistochemical appearance of SK-Br-3 cells incubated for 6 days with *erb*B-2 oligonucleotides. The colour red indicates staining for the erbB-2 oncoprotein, as described in the Material and methods section. Top left: Control cells not treated with oligonucleotides. Top right: Cells treated with sense *erb*B-2 oligonucleotides. Bottom left: Cells treated with nonsense *erb*B-2 oligonucleotides. Bottom right: Cells treated with antisense *erb*B-2 oligonucleotides. The last panel shows a marked reduction of erbB-2 stain.

with 20 μM *erb*B-2 antisense oligonucleotides was approximately 66% that of untreated cells (Table II). The *erb*B-2 protein levels were inhibited in a dose-dependent manner by *erb*B-2 antisense oligonucleotides, with a 50% reduction obtained with 50 μM. Comparatively, the sense oligonucleotides did not decrease *erb*B-2 levels at 50 μM.

We consistently observed enhanced *erb*B-2 staining index in cells treated with sense oligonucleotides. This same effect was seen when a scrambled-sequence nonsense oligonucleotide was used (Figure 3 and data not shown). We were unable to explain this non-specific effect, which did not correlate with oligonucleotide concentration. However, the apparent enhancement of the staining reaction by oligonucleotides suggests that the magnitude of the inhibitory effect of antisense oligonucleotides may be an underestimate of the actual effect.

### Discussion

Our experiments provide evidence that *erb*B-2 overexpression is important for the *in vitro* proliferation of human breast carcinoma cell lines derived from tumours with an amplified *erb*B-2 gene. The decrease in erbB-2 protein levels induced by *erb*B-2 antisense oligonucleotides significantly reduced cellular growth and thymidine incorporation into DNA of these breast cancer cell lines. This antiproliferative effect was specific, since control oligonucleotides, which did not reduce erbB-2 protein levels, had no effect on cellular growth or DNA synthesis. The specificity of the effect of *erb*B-2 antisense oligonucleotides was further demonstrated by their lack of effect on MDA-MB-468 and MCF-7 cells, which do not express or express low levels of *erb*B-2.

*erb*B-2 antisense oligonucleotides did not arrest completely the growth of the cells overexpressing *erb*B-2. In our experiments we observed a maximum of 60% inhibiton of growth. We generally treated our cultures once with oligonucleotides and measured growth at 7 days. Since intracellular degradation of oligonucleotides occurs, a daily treatment may have achieved a greater degree of cell growth inhibition. Furthermore, the cell lines used in these experiments contained other genetic alterations in addition to *erb*B-2 amplification, which also may drive cell growth independent of *erb*B-2. For example, SK-Br-3 cells express c-*fms* and CSF-1 and have an amplification of the c-*myc* gene (Horiguchi *et al*., 1988). MDA-MB-453 and MDA-MB-361 cells overexpress *erb*B-3 (Kraus *et al*., 1989; Plowman *et al*., 1990). BT-474 and MDA-MB-361 cells express oestrogen and progesterone receptors and manifest a growth response to oestradiol treatment *in vitro* and also when grown in nude mide (R. Colomer & E.P. Gelmann, unpublished data).

A third possible explanation for our results is the existence of cell line heterogeneity. Preliminary evidence from 20 clonal derivatives of SK-Br-3 cells shows that the antiproliferative effects of *erb*B-2 antisense oligonucleotides are retained by less than 50% of the clones, and that this is not related to loss of expression of erbB-2 protein. A recent report has suggested that a subline of the lymphoma cells DHL-4 develops resistance to an antisense oligonucleotide by the emergence of intracellular degradation that is absent in the parental line (Ryte *et al*., 1993). Therefore there may be cellular heterogeneity regarding sensitivity to oligonucleotides, and resistance to these agents may be a problem in future experiments. Nevertheless, our finding that *erb*B-2 antisense oligonucleotides inhibit the growth of cells overexpressing *erb*B-2 by 50–60% is consistent with a similar inhibition of proliferation observed in SK-Br-3 cells with erbB-2 antibodies (Hudziak *et al*., 1989).

We have shown that *erb*B-2 antisense oligonucleotides induce a dose-related decrease in erbB-2 protein levels measured by immunohistochemistry in SK-Br-3 cells. A 20 μM concentration of *erb*B-2 antisense oligonucleotide induced a 35% decrease in erbB-2 protein staining. Cells treated with sense *erb*B-2 oligonucleotides, in contrast, did not decrease erbB-2 levels and rather showed an increase in erbB-2 protein staining. The specificity of the effect of antisense oligonucleotides was further substantiated by the fact that we observed no reduction in erbB-2 staining by *erb*B-2 sense oligonucleotides, even at concentrations at which these compounds had non-specific antiproliferative effects. The erbB-2 staining increase induced by *erb*B-2 sense oligonucleotides does not seem to be specific for the sense sequence that we used, since it did not correlate with the concentration of oligonucleotide and, furthermore, other unrelated oligonucleotides induced a similar small increase in erbB-2 staining (data not shown). *erb*B-2 antisense oligonucleotides decreased the levels of erbB-2 protein in the cell membrane without a concurrent increase in intracellular stain, which is consistent with a decreased erbB-2 protein synthesis. This contrasts with the down-regulating effect of the erbB-2 ligand gp30 on cells overexpressing *erb*B-2. gp30 decreases membrane-associated erbB-2, but partly at the expense of increased intracellular accumulation of erbB-2 protein (Bacus *et al*., 1992).

By inhibiting the expression of erbB-2 protein with antisense oligonucleotides we have shown that breast cancer cells with *erb*B-2 amplification are subject to specific growth inhibition. Our results support the hypothesis that *erb*B-2 oncogene amplification confers a growth advantage on cells (DiFiore *et al*., 1987; Hudziak *et al*., 1987). Other procedures that decrease erbB-2 levels interfere with cellular proliferation. The selective pressure provided by very high erbB-2 receptor levels can be reversed using monoclonal antibodies to erbB-2 (Drebin *et al*., 1988; Hudziak *et al*., 1989). In addition, we have reported that elevated concentrations of gp30 are growth inhibitory in cells with elevated expression of *erb*B-2 (Lupu *et al*., 1990). Clinically, *erb*B-2 is overexpressed in 25% of human primary breast carcinomas, and it is correlated with an adverse prognosis. The experimental data strongly suggest that the *erb*B-2 oncogene and its product can be potential therapeutic targets in a significant fraction of breast carcinomas. Therapeutic strategies aimed at the *erb*B-2 gene may have a relatively high therapeutic index. We make this speculation based on the fact that two cancer cell lines without *erb*B-2 overexpression showed no growth effects after *erb*B-2 antisense oligonucleotides treatment and, similarly erbB-2 antibodies and gp30 had no antiproliferative effects on cells without overexpression (Hudziak *et al*., 1989; Lupa *et al*., 1990). This theoretical approach is further supported by the observations that there is homogeneous overexpression of *erb*B-2 among the cancer cells in breast tumours that overexpress the oncogene (Iglehart *et al*., 1990), while the surrounding mammary tissue is essentially void of *erb*B-2 overexpression, as are other normal tissues throughout the body (Press *et al*., 1990). Antisense oligonucleotides chemical analogues synthesised to confer relative serum and cellular nuclease resistance represent a new class of compounds with potential application for gene-specific therapeutic intervention (Tidd, 1990). Experiments with these agents *in vivo* will help define the spectrum of applications for oligonucleotides and their derivatives.

The authors thank C. Sommers, J. Voeller and F. Kern for their help and suggestions, C.R. King (Molecular Oncology, Inc., Gaithesburg, MD) for *erb*B-2 antibodies, A. Khatri (Macromolecular Synthesis and Sequencing Facility, Georgetown University) for the synthesis of fluoresceinated oligonucleotides, J.S. Cohen (Georgetown University Laboratories, Rockville, MD) and J. Zon (Applied Biosystems, Inc., Foster City, CA) for advice and reagents, O. Blair and A. Brown (Georgetown University) for the FACS analysis, S. Byers (Department of Anatomy and Cell Biology, Georgetown University) for his help with the fluorescence microscopy, and D. Miller and C. McDevitt for their excellent assistance. R.C. has been supported by fellowships from the National Cancer Institute/European Organization for Research and Treatment of Cancer (NCI/EORTC) Exchange Program (Brussels, Beglium), the Fondo de Investigaciones Sanitarias (Madrid, Spain), the Programa de Perfeccionamiento de Doctores y Tecnólogos, Comisión Interministerial de Ciencia y Tecnologia (Madrid, Spain), the Cancer Research Foundation of American (Alexandria, Virginia) and by an American Society of Clinical Oncology Young Investigator Award.

### References

BACUS, S.S., BACUS, J.W., SLAMON, D.J. & PRESS, M.F. (1990). HER-2/Neu oncogene expression and DNA ploidy analysis in breast cancer. *Arch Pathol. Lab. Med.*, **114**, 164–169.

BACUS, S.S., HUBERMAN, E., CHIN, D., KIGUCHI, K., SIMPSON, S., LIPPMAN, M. & LUPU, R. (1992). A ligand for the erbB-2 oncogene product (gp30) induces differentiation of human breast cancer cells *Cell. Growth Diff.*, **3**, 401–411.

BOUCHARD, L., LAMARRE, L., TREMBLAY, P.J. & JOLICOEUR, P. (1989). Stochastic appearance of mammary tumors in transgenic mice carrying the MMTV c-*neu* oncogene. *Cell*, **57**, 931–936.

CARTER, G. & LEMOINE, N.R. (1993). Antisense technology for cancer therapy: does it make sense? *Br. J. Cancer*, **67**, 869–876.

COUSSENS, L., YANG-FENG, T.L., LIAO, Y.-C., CHEN, E., GRAY, A., MCGRATH, J., SEEBURG, P.H., LIBERMANN, T.A., SCHLESSINGER, J., FRANCKE, U., LEVINSON, A. & ULLRICH, A. (1985). Tyrosine kinase receptor with extensive homology to EGF receptor shares chromosomal location with *neu* oncogene. *Science*, **230**, 1132–1139.

DI FIORE, P.P., PIERCE, J.H., KRAUS, M.H., SEGATTO, O., KING, C.R. & AARONSON, S.A. (1987). *erb*B-2 is a potent oncogene when overexpressed in NIH/3T3 cells. *Science*, **237**, 178–182.

DREBIN, J.A., LINK, V.C., STERN, D.F., WEINBERG, R.A. & GREEN, M.I. (1985). Down-modulation of an oncogene protein product and reversion of the transformed phenotype by monoclonal antibodies. *Cell*, **41**, 695–706.

DREBIN, J.A., LINK, V.C., WEINBERG, R.A. & GREENE, M.I. (1986). Inhibition of tumor growth by a monoclonal antibody reactive with an oncogene-encoded tumour antigen. *Proc. Natl Acad. Sci. USA*, **83**, 9129–9133.

DREBIN, J.A., LINK, V.C. & GREENE, M.I. (1988). Monoclonal antibodies specific for the *neu* oncogene product directly mediate anti-tumor effects *in vivo*. *Oncogene*, **2**, 387–394.

HÉLÈNE, C. (1989). Artificial control of gene expression by oligodeoxynucleotides covalently linked to intercalating agents. *Br. J. Cancer*, **60**, 157–160.

HOLT, J.T., REDNER, R.L. & NIENHUIS, A.W. (1988). An oligomer complementary to c-myc mRNA inhibits proliferation of HL-60 promyelocytic cells and induces differentiation. *Mol. Cell. Biol.*, **8**, 963–973.

HORIGUCHI, J., SHERMAN, M.L., SAMPSON-JOHANNES, A., WEBER, B.L. & KUFE, D.W. (1988). CSF-1 and c-*fms* gene expression in human carcinoma cell lines. *Biochem. Biophys. Res. Commun.*, **157**, 395–401.

HUDZIAK, R.M., SCHLESSINGER, J. & ULLRICH, A. (1987). Increased expression of the putative growth factor receptor $p185^{HER2}$ causes transformation and tumorigenesis of NIH 3T3 cells. *Proc. Natl Acad. Sci. USA*, **84**, 7159–7163.

HUDZIAK, R.M., LEWIS, G.D., WINGET, M., FENDLY, B.M., SHEPARD, H.M. & ULLRICH, A. (1989). $p185^{HER2}$ monoclonal antibody has antiproliferative effects in vitro and sensitizes human breast tumour cells to tumour necrosis factor. *Mol. Cell. Biol.*, **9**, 1165–1172.

IGLEHART, J.D., KRAUS, M.H., LANGTON, B.C., HUPER, G., KERNS, B.J. & MARKS, J.H. (1990). Increased *erb*B-2 gene copies and expression in multiple stages of breast cancer. *Cancer Res.*, **50**, 6701–6707.

KRAUS, M.H., POPESCU, N.C., AMSBAUGH, S.C. & KING, R.C. (1987). Overexpression of the EGF receptor-related proto-oncogene *erb*B-2 in human mammary tumor cell lines by different molecular mechanisms. *EMBO J.*, **6**, 605–610.

KRAUS, M.H., ISSING, W., MIKI, T., POPESCU, N.C. & AARONSON, S.A. (1989). Isolation and characterization of *ERBB3*, a third member of the *ERBB*/epidermal growth factor receptor family: evidence for overexpression in a subset of mammary tumors. *Proc. Natl Acad. Sci. USA*, **86**, 9193–9197.

KUMAR, R., SHEPARD, H.M. & MENDELSOHN, J. (1991). Regulation of phosphorylation of the c-*erb*B-2/*HER2* gene product by a monoclonal antibody and serum growth factor(s) in human mammary carcinoma cells. *Mol. Cell. Biol.*, **11**, 979–986.

LUPU, R., COLOMER, R., ZUGMAIER, G., SARUP, J., SHEPARD, M., SLAMON, D. & LIPPMAN, M.E., (1990). A ligand for the erbB-2 oncogene interacts directly with the EGF receptor and $p185^{erbB-2}$. *Science*, **249**, 1552–1555.

MCMANAWAY, M.E., NECKERS, L.M., LOKE, S.L., AL-NASSER, A.A., REDNER, R.L., SHIRAMIZU, B.T., GOLDSCHMIDTS, W.L., HUBER, B.E., BHATIA, K. & MAGRATH, I.T. (1990). Tumour-specific inhibition of lymphoma growth by an antisense oligodeoxynucleotide. *Lancet*, **335**, 808–811.

MAGUIRE, H.C. & GREENE, M.I. (1989). The *neu* (c-erbB-2) oncogene. *Semin. Oncol.*, **16**, 148–155.

MELANI, C., RIVOLTINI, L., PARMIANI, G., CALABRETTA, B. & COLOMBO, M.P. (1991). Inhibition of proliferation by c-myb antisense oligodeoxynucleotides in colon adenocarcinoma cell lines that express c-myb. *Cancer Res.*, **51**, 2987–2901.

MORRISON, R.S. (1991). Suppression of basic fibroblast growth factor expression by antisense oligodeoxynucleotides inhibits the growth of transformed human astrocytes. *J. Biol. Chem.*, **266**, 728–734.

MULLER, W.J., SINN, E., PATTENGALE, P.K., WALLACE, R. & LEDER, P. (1988). Single-step induction of mammary adenocarcinoma in transgenic mice bearing the activated c-*neu* oncogene. *Cell*, **54**, 105–115.

PAIK, S., HAZAN, R., FISHER, E.R., SASS, R.E., FISHER, B., REDMOND, C., SCHLESSINGER, J., LIPPMAN, M.E. & KING, C.R. (1990). Pathologic findings from the National Surgical Adjuvant Breast and Bowel Project: prognostic significance of *erb*B-2 protein overexpression in primary breast cancer. *J. Clin. Oncol.*, **8**, 103–112.

PLOWMAN, G.D., WHITNEY, G.S., NEUBAUER, M.G., GREEN, J.M., MCDONALD, V.L., TODARO, B.J. & SHOYAB, M. (1990). Molecular cloning and expression of an additional epidermal growth factor receptor-related gene. *Proc. Natl Acad. Sci. USA*, **87**, 4905–4909.

PRESS, M.F., CORDON-CARDO, C. & SLAMON, D. (1990). Expression of the HER-2/*neu* proto-oncogene in normal human adult and fetal tissues. *Oncogene*, **5**, 953–962.

REED, J.C., STEIN, C., SUBASINGHE, C., HALDAR, S., CROCE, C.M., YUM, S. & COHEN, J. (1990). Antisense-mediated inhibition of *BCL2* protooncogene expression and leukemic cell growth and survival: comparisons of phosphodiester and phosphorothioate oligodeoxynucleotides. *Cancer Res.*, **50**, 6565–6570.

ROSOLEN, A., WHITESHELL, L., IKEGAKI, N., KENNET, R.H. & NECKERS, L.M. (1990). Antisense inhibition of single-copy N-*myc* expression results in decreased cell growth without reduction of c-*myc* protein in a neuroepithelioma cell line. *Cancer Res.*, **50**, 6316–6322.

RYTE, A., MORELLI, S., MAZZEI, M., ALAMA, A., FRANCO, P., CANTI, G.F. & NICOLIN, A. (1993). Oligonucleotide degradation contributes to resistance to antisense compounds. *Anti-Cancer Drugs*, **4**, 197–200.

SAISON-BEHMOARAS, T., TOCQUÉ, B., REY, I., CHASSIGNOL, M., THUONG, N.T. & HÉLÈNE, C. (1991). Short modified antisense oligonucleotides directed against Ha-*ras* point mutation induce selective cleavage of the mRNA and inhibit T24 cells proliferation. *EMBO J.*, **10**, 1111–1118.

SARUP, J.C., JOHNSON, R.M., KING, K.L., FENDLY, B.M., LIPARI, M.T., NAPIER, M.A., ULLRICH, A. & SHEPARD, H.M. (1991). A cell growth inhibitory monoclonal antibody to $p185^{HER2}$ exhibits partial agonistic properties. *Growth Regul.*, **1**, 72–82.

SCHECHTER, A.L., HUNG, M.-C., VAIDYANATHAN, L., WEINBERG, R.A., YANG-FENG, T.L., FRANCKE, U., ULLRICH, A. & COUSSENS, L. (1985). The *neu* gene: an *erb*B-homologous gene distinct from and unlinked to the gene encoding the EGF receptor. *Science*, **229**, 976–978.

SLAMON, D.J., CLARK, G.M., WONG, S.G., LEVIN, W.J., ULLRICH, A. & MCGUIRE, W.L. (1987). Human breast cancer: correlation of relapse and survival with amplification of the HER-2/*neu* oncogene. *Science*, **235**, 177–182.

SZCZYLIK, C., SKOERSKI, T., NICOLAIDES, N.C., MANZELLA, L., MALAGUARNERA, L., VENTURELLI, D., GERWITZ, A.M. & CALABRETTA, B. (1991). Selective inhibition of leukemia cell proliferation by BCR-ABL antisense oligodeoxynucleotides. *Science*, **253**, 562–565.

TAGLIABUE, E., CENTIS, F., CAMPIGLIO, M., MASTROIANNI, A., MARTIGNONE, S., PELLEGRINI, R., CASALINI, P., LANZI, C., MENARD, S. & COLNAGHI, M.I. (1991). Selection of monoclonal antibodies which induce internalization and phosphorylation of $p185^{HER2}$ and growth inhibition of cells with HER2/*NEU* gene amplification. *Int. J. Cancer*, **47**, 933–937.

TANDON, A.K., CLARK, G.M., CHAMNESS, G.C., ULLRICH, A. & MCGUIRE, W.L. (1989). HER-2/*neu* oncogene protein and prognosis in breast cancer. *J. Clin. Oncol.*, **7**, 1120–1128.

TARAKHOVSKY, A.M., RESNIKOV, M., ZAICHUK, T., TUGUSHEVA, M.V., BUTENKO, Z.A. & PRASSOLOV, V.S. (1990). Polymorphic changes of cell phenotype caused by elevated expression of an exogenous NEU proto-oncogene. *Oncogene*, **5**, 405–410.

TIDD, D.M. (1990). A potential role for antisense oligonucleotide analogues in the development of oncogene targeted cancer chemotherapy. *Anticancer Res.*, **10**, 1169–1182.

UHLMAN, E. & PEYMAN, A. (1990). Antisense oligonucleotides: a new therapeutic principle. *Chem. Rev.*, **90**, 543–584.

VAN LEEUWEN, F., VAN DE VIJVER, M.J., LOMANS, J., VAN DEEMTER, L., JENSTER, G., AKIYAMA, T., YAMAMOTO, T. & NUSSE, R. (1990). Mutation of the human *neu* protein facilitates down-modulation by monoclonal antibodies. *Oncogene*, **5**, 497–503.

WICKSTROM, E.L., BACON, T.A., GONZALEZ, A., FREEMAN, D.L., LYMAN, G.H. & WICKSTROM, E. (1988). Human promyelocytic leukemia HL-60 cell proliferation and c-*myc* protein expression are inhibited by an antisense pentadecadeoxynucleotide targeted against c-*myc* mRNA. *Proc. Natl Acad. Sci. USA*, **85**, 1028–1032.

WRIGHT, C., ANGUS, B., NICHOLSON, S., SAINSBURY, R.C., CAIRNS, J., GULLICK, W.J., KELLY, P., HARRIS, A.L. & HORNE, C.H.W. (1989). Expression of *erb*B-2 oncoprotein: a prognostic indicator in human breast cancer. *Cancer Res.*, **49**, 2087–2090.

YAMAMOTO, T., IKAWA, S., AKIYAMA, T., SEMBA, K., NOMURA, N., MIYAJIMA, N., SAITO, T. & TOYOSHIMA, K. (1986). Similarity of protein encoded by the human *erb*B-2 gene to epidermal growth factor receptor. *Nature*, **319**, 230–234.